# Characterizing the Impact of Social Inequality on COVID-19 Propagation in Developing Countries

**DOI:** 10.1109/ACCESS.2020.3024910

**Published:** 2020-09-18

**Authors:** Evelin Helena Silva Cardoso, Marcelino Silva Da Silva, Francisco Eguinaldo De Albuquerque Félix Júnior, Solon Venâncio De Carvalho, André Carlos Ponce De Leon Ferreira De Carvalho, Nandamudi Vijaykumar, Carlos Renato Lisboa Francês

**Affiliations:** 1 Postgraduate Program in Electrical EngineeringFederal University of Pará (UFPA) Belém 66075110 Brazil; 2 Computer Science AreaFederal Rural University of the Amazon (UFRA) Capitão Poço 68650-000 Brazil; 3 National Institute for Space Research (INPE) São José dos Campos 12227010 Brazil; 4 Department of Applied Mathematics and StatisticsUniversity of São Paulo (USP) São Carlos 13566590 Brazil; 5 Institute of Science and Technology, Federal University of São Paulo (UNIFESP) São José dos Campos 12247014 Brazil

**Keywords:** COVID-19~propagation, social inequality, non-pharmaceutical interventions, developing countries

## Abstract

The world faces a pandemic not previously experienced in modern times. The internal mechanism of SARS-Cov-2 is not well known and there are no Pharmaceutical Interventions available. To stem the spread of the virus, measures of respiratory etiquette, social distancing and hand hygiene have been recommended. Based on these measures, some countries have already managed to control the COVID-19 propagation, although in the absence of pharmaceutical interventions, this control is not definitive. However, we have seen that social heterogeneity across populations makes the effects of COVID-19 also different. Social inequality affects the population of developing countries not only from an economic point of view. The relationship between social inequality and the health condition is not new, but it becomes even more evident in times of crisis, such as the one the world has been facing with COVID-19. How does social inequality affect the COVID-19 propagation in developing countries is the object of this study. We propose a new epidemic SEIR model based on social indicators to predict outbreak and mortality of COVID-19. The estimated number of infected and fatalities are compared with different levels of Non-Pharmaceutical Interventions. We present a case study for the Deep Brazil. The results showed that social inequality has a strong effect on the propagation of COVID-19, increasing its damage and accelerating the collapse of health infrastructure.

## I. Introduction

After its outbreak in China (Wuhan) in December 2019, COVID-19 has affected the entire world. The World Health Organization (WHO) declared its outbreak as a pandemic in March 2020 [1]. Four months after the pandemic was declared more than 8 million people have been confirmed infected with the disease accumulating more than 400,000 deaths worldwide [2]. This uncontrolled spread draws international attention, due to its devastating impact on health care systems. These systems can be overloaded, leading to eventual collapse. Therefore, decision making tools, based on mathematics and artificial intelligence, are required to assist the authorities to concentrate efforts to flatten the epidemic curve so that there is relief in the healthcare infrastructure. When Pharmaceutical Interventions through vaccines or accepted medication are not yet available, the so called Non-Pharmaceutical Interventions (NPIs) play a significant role in influencing the trajectory of the epidemic.

World Health Organization recommends personal protective and environmental measures, such as respiratory etiquette, social distancing, face masks, hand hygiene and surface and object cleaning to reduce transmission as well as the total number of infections and severe cases [3]. It is also necessary to isolate sick individuals, quarantine the exposed individuals and advise the population to stay at home in order to reduce the rate of contamination. This is recommended by experts in epidemiology, who warn that only when the reproduction number ($R_{0}$) is below 1, the growth in the number of cases and deaths begins to slow down. Therefore, NPIs can drastically reduce the escalation of the propagation and may relieve the impact on the healthcare infrastructure.

COVID-19 does not affect everyone equally. It magnifies pre-existing class differences and reveals a social gap among individuals living in the same country, even in the same city, but experiencing completely different living conditions. The main issue is the high concentration of people in precarious living conditions, with high demographic density per room and inadequate basic sanitation services. Another reality is that, in underdeveloped countries, a significant proportion of workers classified as essential services (not considering health professionals, such as medical doctors), predominantly occupy low-income jobs.

In the report “Calibrating long-term non-pharmaceutical interventions for COVID-19: principles and facilitation tools” [3], WHO brings guiding principles to assess health system capacity to manage cases and to balance those assessments against the epidemiological and socioeconomic impacts of NPIs, including the protection of vulnerable populations. World Health Organization emphasizes specific ways in which NPIs impact on vulnerable populations should be considered and mitigated whenever possible, including loss of income, reduced access to health and other essential services, increased social isolation and inability to self-isolate in crowded living conditions.

With respect to the reality of precarious conditions of habitation that is very high in Brazil, there has been a long discussion on the conditions of basic sanitation. This discussion led the Brazilian Senate to approve a bill “New Legal Framework for Basic Sanitation”. One of the main points discussed and approved in this bill was the possibility of privatization of the basic sanitation services by State Governments. It is expected that the new legal framework for sanitation will guarantee millions of Brazilians access to water and sewage treatment services [4].

According to the current data raised by the Brazilian Senate for debate on the subject, up to 6 thousand newborns die each year from diseases related to the lack of basic sanitation, 35 million Brazilians do not have access to drinking water and more than one hundred million Brazilians, almost half of the population, do not have access to the sewage network. In general terms, 35.7% of the population does not have access to any basic sanitation service, which corresponds to approximately 75 million Brazilians [5]. The global report presented by the United Nations Children’s Fund (UNICEF) pointed out that 40% of people do not have access to water and soap [6]. Hence, many Brazilians are unable to meet the recommendations regarding prevention measures related to the expansion of hygiene practices.

In the peripheries of Brazilian cities, it is common to find houses with only few rooms, without ventilation, where the space is usually shared by several people (for example, elderly people living with adults and children). Housing has been recognized as an important determinant factor of health. Crowded housing increases the risk of exposure to infectious disease and stress [7]. Also, overcrowding can result in higher infection rates [8]. In this context, household crowding is a condition where the number of occupants exceeds the capacity of the dwelling space available, whether considered as rooms, bedrooms or floor area [7]. WHO Housing and Health Guidelines report [7] also presents a table with some metrics on crowding, which may vary from country to country. For example, according to the American Crowding Index, crowding occurs if there is more than one person per room; severe crowding occurs if there are more than 1.5 persons per room (excluding bathrooms, balconies, porches, foyers, hall-ways and half-rooms) [9]. According to the Argentinian National Institute of Statistics and Censuses [10] and the United Nations – Habitat [11], households with critical overcrowding are considered those with more than three people per room (excluding the kitchen and bathroom).

Regarding the high density of people per room, in Brazil about 6% of the population, 11.5 million people, live in households with three or more people per room. This makes it difficult to comply with home quarantine measures, when someone in the household is ill. It is not feasible to avoid contagion with other family members, disabling social isolation as contacts of family members with neighbors or others are inevitable, even not living together. The concentration of these households is higher in the metropolitan regions, for example, 8.8% of the population in the Metropolitan Region of São Paulo, 7.1% of the population in the Metropolitan Region of Rio de Janeiro and 11% of the population in the Metropolitan Region of Belém (largest in the North of the country) lives in households with this characteristic. It is important here to clarify that the term bedroom refers to any room within the house that serves for sleeping purposes. In countries such as Brazil and India, there are houses with only one single room or few rooms. Even living rooms and many a time, kitchens are also used for sleeping purposes, thus making too difficult to isolate infected individuals.

These data were calculated from the National Household Sample Survey (PNAD) of the Brazilian Institute of Geography and Statistics (IBGE) [12]. The Brazilian Institute of Geography and Statistics is an entity of the federal public administration, linked to the Ministry of Economy and is the main provider of geographic, social, economic and demographic data and information about Brazil. The National Household Sample Survey is a survey carried out by IBGE in a sample of Brazilian households, in order to determine general characteristics of the population, including socioeconomic data, such as population, education, work, income, housing, among others. Regarding occupation, according to the report “Sociodemographic profile of favela residents with UPP (Pacification Police Unit) in the city of Rio de Janeiro in 2016” released by the Center for Security and Citizenship Studies [13], the 12 most frequent occupations of the population of 37 territories with UPP in the municipality of Rio de Janeiro, includes various service sector jobs (waiter, elevator operator, street sweeper, cleaning, doorperson, delivery persons, etc.), domestic work in third-party houses, merchants, attendants, drivers, among others. Therefore, for many of these professionals, stopping work is not an option, even in the midst of a pandemic. In Brazil, the term “favela” is related to a type of low-income slum neighborhood. The National Household Sample Survey (2018) found that 50% of the Brazilian population, approximately 104 million people, live on R$413 per capita per month [12], equivalent to $ 79.12 (quoted on July 27, 2020).

The unprecedented survey “Pandemia in the Favela - The reality of 14 million people in the fight against the new Coronavirus” [14] carried out by Data Favela, a partnership of the Locomotiva Institute, the Central Única das Favelas (CUFA) and the Favela Holding, provides an insight into the time of the pandemic, pointing out, among other data, how is the perception of the favela residents about the evolution of the pandemic. According to the survey, around four to seven people live in 48% of households in these communities. However, 59% of homes have a maximum of two bedrooms. In the general average, 2.6 people sleep per room, making the isolation of persons infected or suspected of infection impractical at home. The survey also points out that more than half of favela residents, 51%, are unable to follow the recommended measures to prevent the spread of the new coronavirus (COVID-19).

All of these data reflect the high degree of social inequality in Brazil. Besides, according to the IBGE surveys, from the average real monthly income usually received from all types of labor considered for the period 2012 to 2019, in the period 2012 to 2015 there was a decrease in inequality measured by the Gini index, reaching the lowest value of 0.494. Since then, inequality has been increasing, reaching the Gini index value of 0.509 in 2019. This index represents the situation in which 1% of the population with the highest income received, on average, R$28,659 per month (equivalent to $ 5,490), while half of the population with the lowest income earned R$850 (equivalent to $ 162), corresponding to a difference of 33.7 times [15] (quoted on July 27, 2020).

In addition, many of these individuals use public transport to reach their workplaces, further increasing their exposure rate as they have to interact with many other individuals throughout the day and return at the end of the day to their homes, also exposing their kin. With these data, it is possible to state that the chances of facing the high transmissibility of COVID-19 in countries with high social inequality, such as Brazil, are low.

In this study, social inequality refers to situations implying some degree of social imbalance, that systematically puts some groups at a disadvantage in relation to some opportunities, such as to stay healthy or have access to health services in the midst of a pandemic. According to the study presented in [16] which provides an overview of socioeconomic inequalities for a large number of non-communicable diseases in 20 European countries for women and men, the more socially and economically disadvantaged a person is, the more likely he/she is to suffer from high blood pressure, diabetes and heart or respiratory diseases. Another aggravating factor is that people in unfavorable socio-economic circumstances can also be more exposed to infections due to precarious living conditions. These aspects of inequality are typical of developing countries, since they negatively affect socio-economic indicators globally used for country classification.

In [17], the author addresses among other topics, social inequalities in Health and the reflexes of an individual’s social position in their health. Different populations attach greater or lesser importance to health as a fundamental human right. The author highlights the political behavior of most leaders, especially, in European countries, who increasingly attach importance to reduce social inequalities in health, considering that national health systems and other social policies must have as their main objective to achieve equity. On the other hand, in countries where welfare state regimes are more prominently liberal, as in the United States, there is a minimal welfare from the state and great dependence on the private sector [8]. For example, according to the Kaiser Family Foundation [18], that provides critical information on health coverage in the United States of America, the private sector is the main source of health coverage in United States (US). So, the right to health is intrinsically related to individual ability to financially support appropriate services.

In spite of the United States being a developed country, it also has a high degree of social inequality. The report “COVID-19 exacerbating inequalities in the US”, by Aaron van Dorn, Rebecca E Cooney, Miriam L Sabin [19], confirms existing disparities, within New York City and other urban centers. African American and other colored communities have been especially affected by the pandemic. Besides, across the country, deaths due to COVID-19 are disproportionately high among African Americans compared with the overall population. The authors justify that part of such a disproportionate impact of the COVID-19 pandemic on colored communities in the US has been structural factors that prevent those communities from practicing social distancing. Also, minority populations in the United States disproportionately are in fact the workforce of essential activities. Hence, they have no privilege of staying protected at home.

Despite its limitations and difficulties, in Brazil, healthcare is a right. Even a non-resident of the country can have access to this system free of cost. The universal health system in Brazil is called the Unified Health System (SUS). The Unified Health System is decentralized, so that the management, formulation and implementation of policies are the responsibility of state and municipal governments. Brazil’s health system is financed through a variety of taxes at the federal, state and municipal levels and has been crucial in combating the COVID-19 epidemic.

In order to analyze the correlation of the main socioeconomic variables (related to Human Development Index, housing, sanitation) with the dissemination of COVID-19 in Brazilian capitals we developed a correlation matrix using Pearson’s correlation coefficient [20]. It has a value between +1 and −1, where 1 is total positive linear correlation, 0 is no linear correlation, and −1 is total negative linear correlation. The study includes Brazilian capitals that have already registered 75 days after their first case. The used indicators were extracted from the online repositories of Brazilian Institute of Geography and Statistics (IBGE) [21] and Atlas Brasil [22], that use results from the National Research based on Domicile Sampling (PNAD) and demographic census. Information related to COVID-19 was acquired on the Brasil.io website [23] which performs a web scraping in the daily reports released by the health departments of the Brazilian states.

It is possible to observe a moderate negative correlation of the Human Development Index (HDI) - and the variables that compose it: income, longevity and education - with cases and deaths for groups of 100,000 inhabitants, indicating that, possibly, the municipalities with the worst HDIs tend to present more severe pictures in the impacts caused by the disease. In addition, by assessing household issues such as basic sanitation or population density per bedroom, it is possible to note again the existence of a moderate correlation, assuming that people without running water, without access to bathrooms, with inadequate water and sewage or using dorms with more than two people are more likely to spread the disease. At the same time, the matrix also presents interesting indicators for cases and deaths per 100,000 inhabitants in relation to the labor market, especially for informal workers, who, possibly, tend to be more vulnerable to COVID-19 in Brazilian capitals. The data show that there is a correspondence between social and economic indicators with the spread of the disease.

To contribute to the current public health crisis our attempt in this study is to develop a system to estimate the future behavior of COVID-19 considering the social inequality typical of developing countries in order to show its impact on the propagation. Despite many developing countries have a median level of development, dynamic industrial activity, a good capacity to export as well as economic growth, such as Brazil, Mexico and India, in this work we consider especially countries that suffer from social inequality due to the improper distribution of income and wealth, low education levels, habitation problems (overpopulated domiciles without proper sanitation conditions), malnutrition, high unemployment and lack of basic infrastructure (treatment of water and sewage).

In the context of COVID-19, developing countries may have a devastating impact because of: (i) domiciles in precarious areas and overpopulated houses with few rooms; and (ii) low access to clean water and basic sanitation. The main impact is related to inability to follow the recommendations of social distancing and hygiene. Thus, a part of the population of these countries is, indeed, unable to follow such recommendations in spite of several efforts, campaigns, advertisements, etc. We believe that this picture may intensify the scenario by spreading the disease leading to collapse of health infrastructure.

Therefore, we describe a new epidemic model based on the SEIR model [24], more realistic for developing countries. We included to SEIR an important factor considering part of infectious people will continue infecting others even when in home quarantine. This factor quantifies the effect of lack of basic sanitation and access to clean water and high density of people per room in the studied region. Our objective is to offer to developing countries, such as Brazil, a most appropriate model to estimate the impact of social inequality on the propagation of COVID-19 and its effect on healthcare infrastructure. This will enable stronger grounds for the decision makers to implement mechanisms to properly respond to this crisis.

It is also noteworthy that, in addition to the main objective mentioned above, this article also demonstrates how much social conditions can influence the spread of the disease and that, therefore, new models that consider such social conditions of the population should be used for the adequate magnitude and planning of measures to combat the pandemic. The paper has seven sections. Section I introduces this study, Section II discusses studies related to this work, Section III presents the new epidemic model for characterizing the impact of social inequality on COVID-19 propagation in developing countries, Section IV describes the adopted methodology, Section V shows the main results, Section VI presents the limitations of the study and section VII summarizes the paper and finalizes this text with the main conclusion.

## II. Related Work

Monitoring and predicting emerging infectious diseases is vital to the society in order to support the decision making by authorities to manage the public health crisis. COVID-19 pandemic is not different. Specialists have employed mathematical models that represent the dynamics of the epidemics over time, for example, SIR (Susceptible, Infectious, Recovered) and SEIR (Susceptible, Exposed, Infectious, Recovered) models [24]. SIR and SEIR are compartmental models, used mainly to model the spread of infectious diseases over populations. The SIR model describes the flow of individuals within a considered population through three mutually exclusive stages of infection: susceptible, infected and recovered. The SEIR model is derived from the SIR model, which uses as parameters a contamination rate ($\beta $) and a recovery/removal rate ($\gamma $) [24]. SEIR adds a latent period ($\sigma $) to the SIR model. During this period, the number of pathogens is very low to actively transmit the infection to Susceptible individuals. Hence, in this model, the Exposed state includes the individual that is infected but not yet infectious.

In the COVID-19 context, one important and relevant contribution was proposed in [25]. Epidemic size was determined from the age-structured SEIR. The model is based on contact patterns, household size and demographics and income status of different countries. Demand for healthcare was estimated from hospitalization and fatality rates, obtained from analysis on China. They estimated that lack of interventions would have infected 7 billion and killed 40 million worldwide.

A simplified SIR model was used to study an early prediction of the epidemic of the 2019-nCoV in Mainland China [26]. Considering all the sensitivity experiments with respect to infection rate and removal rate, they conclude that the inflection point of 2019-nCoV would occur in late February to early March. They also emphasized the importance of the openness and transparency in releasing the data relevant to the public health to improve the accuracy of estimates. In [27], the researchers proposed a SIR model including dead individuals, called SIRD model, to provide estimations of the basic reproduction number ($R_{0}$), and the daily infection mortality and recovery rates. A case study for Hubei, China was performed. They also considered scenarios in which the real number of infected individuals is much higher than the official numbers, especially because of those with asymptomatic or mild courses.

The researchers in [28] developed a mathematical model called $\theta $-SEIHRD, which takes into account the effect of undetected infected people, on the COVID-19 spread. They denoted by $\theta $ the fraction of detected cases over the real total infected cases. They also incorporated into the model the effect of different sanitary and infectiousness conditions of hospitalized people (differentiating those with mild and severe conditions that will recover from those who will die) and the estimation of the needs of beds in hospitals. They applied the model to the particular case of China. The outputs returned by the simulation fitted quite well the data reported by the WHO for the date and magnitude of the peaks, the number of new cases, new deaths and amount of hospitalized people. Important results were estimated about the impact of the undetected cases on the COVID-19 propagation. They calculated that undetected cases in China could represent around 52% of the total number of estimated cases (168,100 people) and they may have caused around 37% of the total infections. Finally, they also concluded that the magnitude of the epidemic can be drastically reduced when increasing the percentage of detection of cases.

The report [29] attempts to estimate the hidden number of infections currently alive in the population using confirmed cases. For the authors, a hidden case is an undocumented case (an unconfirmed case). An important result indicated an estimate of existing infections at about 10-50 times the new cases confirmed daily in the United States. The authors also establish the relationship between the infection rate and the test rate to bring the epidemic under control, which says that the test rate must accompany the infection rate to prevent an outbreak. This relationship is significant in discussions about the reopening of companies and schools around the world.

In the work [30] we found a compartmental model called SIDARTHE to predict the epidemic evolution for the COVID-19 that discriminates between infected individuals depending on whether they have been diagnosed and on the severity of their symptoms. The model considers eight stages of infection: susceptible, infected, diagnosed, ailing, recognized, threatened, healed and extinct. They compared simulation results with real data on the COVID-19 epidemic in Italy. The results obtained emphasized the importance of restrictive social-distancing measures combined with widespread testing and contact tracing to deal with the ongoing COVID-19 pandemic.

An agent-based model, IndiaSim project, evaluated economic aspects of the Indian healthcare in [31]. The authors employed it together with data from cases in China and Italy to predict infections and hospitalizations in India. Positive aspects, like young population and seasonality, together with negative aspects, like consuming fast food by the young population and social distancing difficulty make this prediction difficult.

The study in [32] shows that, in addition to mathematical models, models based on Machine Learning (ML) can anticipate perioperative outcomes to improve the decision making on the future course of actions. The study demonstrated the capability of ML-induced models to forecast the number of upcoming patients affected by COVID-19 which is presently considered as a potential threat to mankind. Four standard forecasting models were tested: Linear Regression (LR), Least Absolute Shrinkage and Selection Operator (LASSO), Support Vector Machine (SVM) and Exponential Smoothing (ES). The results proved that the ES performed best in the current forecasting domain given the nature and size of the dataset. LR and LASSO also performed well for forecasting to some extent to predict death rate and confirmed cases.

Some experts are studying whether environmental variables have an effect on the spread of the virus. For example, the authors in [33] evaluated the effect of meteorological conditions (temperature, solar radiation, air humidity and precipitation) on 292 daily records of cumulative number of confirmed COVID-19 cases across the 27 Brazilian state capital cities during the 1st month of the outbreak, while controlling for an indicator of the number of tests, the number of arriving flights, population density, proportion of elderly people and average income. The results showed that the number of confirmed cases was mainly related to the number of arriving flights and population density, increasing with both the factors. However, after accounting for these effects, the disease was shown to be temperature sensitive. Their best estimate indicated that a 1°C increase in temperature has been associated with a decrease of 8% in confirmed cases.

As Brazil is an epicenter for COVID-19 in Latin America and one of the most affected countries, it has attracted the attention of the world. The authors in [34] estimated the number of COVID-19 cases and reproduction number in the country. They used a semi-mechanistic Bayesian model of COVID-19 transmission, calibrated using data on reported deaths at the state level, to infer the epidemiological characteristics of the epidemic in Brazil. The results suggested an ongoing epidemic in which substantial reductions in the average reproduction number have been achieved through non pharmaceutical interventions but have not been stringent enough to reduce the reproduction number to less than 1. The results also revealed extensive heterogeneity in predicted attack rates between states. Therefore, the situation is not yet under control and may even become worse with an untimely opening of activities.

The authors in [35] searched for factors affecting contagion, mortality, and the time span between a country’s virus inception and first death. They also analyzed the development of social media and financial markets. For this, they applied a panel data set on all of the countries across the globe from December 31 2019 until March 31 2020 to investigate the patterns of epidemic development. They constructed classification tree regression, panel, and cross-sectional regression models. The main results showed that the speed of severity and contagion differ depending on the continent and on the country. Similarly, financial markets and social media also respond differently to factors affecting contagion and severity. In addition to NPI measures, contact tracking and monitoring of suspected cases also contribute to disease control. The authors in [36] emphasize that mobile phone data can provide access to population estimates and mobility information to enable stakeholders across sectors to better understand COVID-19 trends and geographic distribution. Besides, the data enable new predictive capabilities and allow stakeholders to assess future risks, needs, and opportunities, such as to determine which, whether, and how various interventions affect the spread of COVID-19. The researchers reinforce that when fighting an epidemic like that the world is going through, it is important to understand how lifting and reestablishing various measures translate into behavior, especially to find the optimal combination of measures at the right time and to balance these restrictions with aspects of economic vitality across the COVID-19 pandemic lifecycle.

In this context, researchers evaluated the smartphone contact tracing technology effectiveness and determined the impact of contact tracing precision on the spread and control of COVID-19 [37]. The results showed that, in order to be effective for the COVID-19 disease, the contact tracing technology must be precise, contacts must be traced quickly, and a significant percentage of the population must use the smartphone contact tracing application. Besides, in this sense, in the work [38] we found a stochastic transmission model parameterized to the COVID-19 outbreak in which the authors quantified the potential effectiveness of contact tracing and isolation of cases in controlling COVID-19. In most scenarios studied, the authors concluded that highly effective contact tracing and case isolation were enough to control a new outbreak of COVID-19 within 3 months.

An interesting work is presented in [39]. The authors presented an innovative approach for tracking individuals who have interacted with people showing symptoms, allowing them to warn those in danger of infection and to inform health researchers about the progression of contact-induced diseases. For such, the authors used a multi-disciplinary approach combining techniques from generative machine learning, signal processing, and agent-based modeling to reveal the dynamics of the whole organization or community to track the spreading of flu with mobile phones using partial observations of proximity and symptoms. A case study was applied to a network of a 3000-people university campus and mobilized 300 volunteers. The results showed that it is possible to predict common cold and flu infection two weeks ahead of time with higher level of precision in comparison to a random-guess model.

**FIGURE 1.**
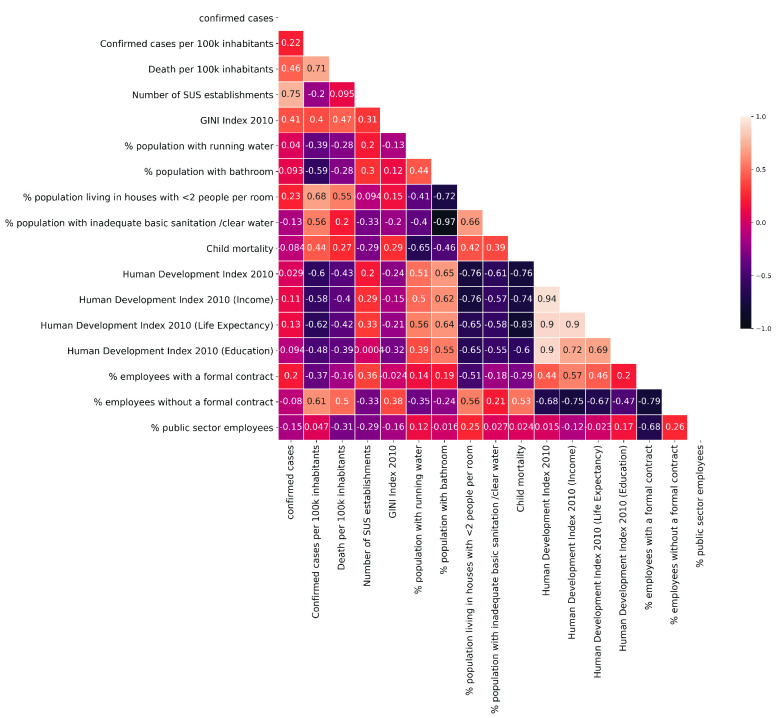
Correlation Matrix of socioeconomic indicators and COVID-19 in Brazil.

Therefore, this paper differs in that it specifically characterizes how social inequality impacts on COVID-19 propagation. For this, we consider housing conditions and access to adequate sanitation services in the proposed model. This choice is justified by the correlation matrix presented in Fig. 1. Additionally, as an example, we use a real scenario to evaluate the model: a case study considering the Deep Brazil, a reality far away from the most central locations. People from this part do not live in big capitals, but in the countryside of Brazil. We estimate the outbreaking from different levels of NPI and its effects in minimizing the impacts of the virus.

In our approach, the average effect of long-term non-pharmaceutical interventions for COVID-19 propagation involving the whole population is captured. This effect is incorporated into our model in an averaged reduction rate of the contamination rate over time to produce long-term estimates and enable the analysis of the impact of social inequality on the worsening of the spread of COVID-19.

## III. Epidemic Model for Characterizing the Impact of Social Inequality on COVID-19 Propagation in Developing Countries

Since the beginning of the COVID-19 pandemic, many models have been proposed to estimate the spread of the virus throughout the population. To the best of our knowledge, it is the first time that a model has inserted socioeconomic indicators in terms of housing conditions and access to basic sanitation services. In this section, we introduce our model for characterizing the impact of social inequality in the COVID-19 propagation. Our proposal is based on the classical SEIR model to develop a new model, including socioeconomic features of regions (countries) presenting a high degree of social inequality. The model will be used for analyzing the impact of COVID-19 outbreak on the regional healthcare infrastructure and to predict the efficacy of different levels of NPIs.

### A. The SEIR Model

The SEIR (Susceptible, Exposed, Infectious, Recovered) model [24] is represented by the following ordinary differential equations, which have to be solved, without considering births and deaths:

\begin{align*} \frac {dS(t)}{dt}=&\frac {-\beta S(t) I(t)}{N}\tag{1}\\ \frac {dE(t)}{dt}=&\frac {\beta S(t) I(t)}{N} - \alpha E(t)\tag{2}\\ \frac {dI(t)}{dt}=&\sigma E(t) - \gamma I(t)\tag{3}\\ \frac {dR(t)}{dt}=&\gamma I(t)\tag{4}\end{align*}

These equations refer, respectively, over time $t$, to the number of individuals susceptible, exposed, infectious and removed. Removed includes individuals that became immune after recovery or died due to the disease. $N$ is the total population of the considered region ($S + E + I + R$). In a simple way, the parameter $\beta $ is considered the contamination rate and can be expressed as the product of daily contact rate ($\eta $) and disease transmission probability ($\theta $) (5) [30]:

\begin{equation*} \beta = \eta \theta\tag{5}\end{equation*}

The parameter $\alpha $ is known as incubation rate (since $1/\alpha $ is the mean incubation period). While the parameter $\gamma $ is known as recovery rate (since $1/\gamma $ is the average duration of recovery or average infectious period).

### B. Our Proposal

Despite the recommendation of house quarantine, for those living in precarious housing conditions (with high demographic density per room of residence and inadequate basic sanitation services), it is highly unlikely for following such recommendation. Moreover, the considered populations live in neighborhoods where other domiciles also have similar conditions. Besides, most of the individuals within this category continue with their labor activities and may be contaminated by others living in healthy conditions and may contaminate their peers when they come back home. Considering these observations, we assume the model should consider that a percentage of the population, even at home quarantine, cannot reduce their contact rate.

**FIGURE 2.**
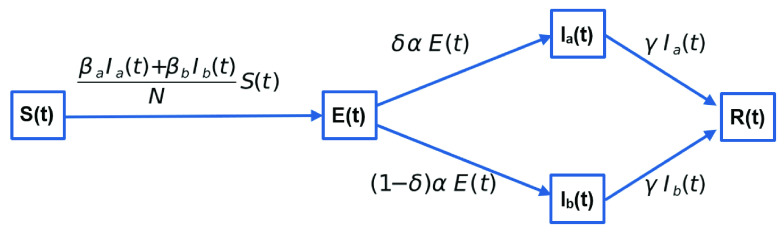
The new epidemic model for characterizing the Impact of Social Inequality on COVID-19 propagation.

To characterize the impact of social inequality on epidemic of COVID-19 propagation in developing countries, we modify the classical SEIR by separating the Infectious compartment as two distinguished compartments depending on the probability of an infectious individual reducing or not her/his rate of contamination, as illustrated in Figure 2. So, compartment $I$ of the classic model is replaced by two new compartments: $I_{a}$ (infectious individuals that can reduce their contamination rate, $\beta _{a}$, as per restrictions advised by health agents) and $I_{b}$ (infectious individuals that cannot effectively reduce their contamination rate, $\beta _{b}$).

Control measures such as quarantine, lockdown, social isolation, use of a mask and hand hygiene reduce these contamination rates. Moreover, these measures reduce both the probability of transmission ($\theta $) and the average number of daily contacts with other people ($\eta $) and, therefore, minimizing the contamination rate ($\beta $). However, due to the issues of social inequality discussed in this article, the effective reduction of the contamination rate only occurs for people in group $a$.

The new model resolves the following system of ordinary differential equations. The notation is presented in Table 1.

\begin{align*} \frac {dS(t)}{dt}=&\frac {-S(t) \beta _{a} I_{a}(t) - S(t) \beta _{b} I_{b}(t)}{N}\tag{6}\\ \frac {dE(t)}{dt}=&\frac {S(t) \beta _{a} I_{a}(t) + S(t) \beta _{b} I_{b}(t)}{N} - \alpha E(t)\tag{7}\\ \frac {dI_{a}(t)}{dt}=&\delta \alpha E(t) - \gamma I_{a}(t)\tag{8}\\ \frac {dI_{b}(t)}{dt}=&(1 - \delta) \alpha E(t) - \gamma I_{b}(t)\tag{9}\\ \frac {dR(t)}{dt}=&\gamma I_{a}(t) + \gamma I_{b}(t)\tag{10}\end{align*}

**TABLE 1:** Notation Table

Symbol	Description
$N$	total population of the considered region
$\delta $	percentage of N that can effectively reduce its rate of contamination
$\alpha $	incubation rate
$\beta _{a}$	rate of contamination for those in group $a$
$\beta _{b}$	rate of contamination for those in group $b$
$\gamma $	recovery rate
$\omega $	rate of cases requiring ICU bed
$S(t)$	number of susceptible individuals to the contagion over time
$E(t)$	number of exposed individuals over time
$I_{a}(t)$	number of infectious individuals in group $a$ over time
$I_{b}(t)$	number of infectious individuals in group $b$ over time
$R(t)$	number of individuals that completed the cycle of COVID-19, due to recovery or death, over time

The number of deaths over time is determined as a percentage of $R(t)$, based on reports on the lethality rate. The number of required hospital beds in intensive care unit (ICU) is calculated as a percentage of the value of peak number of the total Active Infectious over time, $I(t) = I_{a}(t) + I_{b}(t)$, according to the reports observed in other countries.

Observe that an ideal model, besides the contamination rate being different to each group, we would have different incubation, recovery rates and the need to ICU for each group in order to adjust the model. However, from the available publications, it is not possible to determine how different are these rates for the two groups $a$ and $b$. For $\beta _{a}$ and $\beta _{b}$ the difference between the values is due to behavior of the individuals. There are those that can accept the measures to fight the disease and others cannot. For the remaining parameters, it is necessary to possess knowledge on the clinical evolution of the disease. However, at the moment it all depends on how severely they are infected and how their body’s immune system responds. However, studies so far show that some severely affected people may be infectious for longer periods. Individuals who are more susceptible to severe diseases are the elderly (>65 years of age) and people with underlying diseases [1], [40]. Underlying diseases include hypertension, respiratory system disease and cardiovascular disease [41]. As underlying diseases usually attack both groups ($a$ and $b$) and given that in our study the older population is not highlighted, we chose to use the same recovery rate for both groups.

Also, from the literature, we observed other studies based on models that stratify the population by age group, they consider the same incubation and recovery period and that stratification in general influences rates of contact between different individuals across different ages, outbreaks and the size of the epidemic, such as the ones found in [25], [42], [43] and [44]. So, we understand that the proposed model is in line with other models used for the same objective. Even though we do not distinguish the parameters to come up with an ideal model, the proposed model already has significant contributions to better understand the propagation of the disease in places with a high degree of social inequality.

## IV. Methodology

The proposed methodology includes three stages: (i) the acquisition and analysis of data; (ii) the parameter estimation; and (iii) the forecast for the epidemic of COVID-19.

### A. The Acquisition and Analysis of Data

To implement the outbreak prediction model, data from various sectors need to be collected and analyzed. For example, demographic and social factors, local/government records (data describing the distribution and spread of infection through space and time), disease data (including the latent and infectious periods, etc.).

The acquisition of socioeconomic data depends on the availability and transparency of this type of data in each country. Generally, each country has its own Data and Information provider, which coordinates and consolidates statistical information about the nation, in addition to producing and analyzing geographic information.

It is also necessary to acquire data on the evolution of the COVID-19 epidemic in the region under consideration, in terms of confirmed cases and deaths.

Using the socioeconomic data, an analysis must be carried out to measure and to understand the correlation between the social inequality consequences and the disease propagation. Thus, from this correlation study, the main socioeconomic factors can be observed and the percentage of the population that can effectively reduce its rate of contamination can be computed.

For the case of Brazil, which is the case study of this article, it was observed that the main socioeconomic factors that contribute to the spread of SARS-Cov-2 are related to housing conditions, more specifically, the two main factors are the high number of people per bedroom and lack of water supply (Figure 1).

However, for other countries, or for municipalities within the country under study, other factors may better show how much social inequality is influencing the spread of the disease, for example, factors related to education, job characteristics or access to information. Thus, these factors should be used to compute the percentage of the population with the greatest difficulty in effectively adopting protective measures.

Thus, after a general assessment of the socioeconomic factors that influence the spread of the disease in the country under analysis, for the municipality or locality that will be the case study, it is necessary to assess whether the parameters, selected to classify and account for the population in the group that is unable to effectively reduce its rate of contagion, are adequate for the model to be in line with the reality of that municipality.

For the case of Brazil, adherence was assessed for the 27 capitals (26 states and the Federal District). It was based on the comparison between the percentage of the population residing in households with more than two persons per bedroom and the estimated total number (including asymptomatic and people who did not seek health care) of contaminated per 100 thousand inhabitants, which was calculated according to the number of reported deaths per 100 thousand inhabitants and the estimated mortality rate for each state [45].

Table 2 presents in the first column the capitals of the states ordered by the estimate of the total number of contaminated per 100 thousand inhabitants on the 75th day after the first confirmed case. The second column presents the state capitals ordered by the percentage of the population residing in households with more than two per bedroom.

**TABLE 2:** Comparison Between the Percentage of thE Population Residing in Households With More Than Two People Per Bedroom and the Estimated Total Number of Contaminated Per 100 Thousand Inhabitants

State capitals ordered by confirmed cases per 100 thousand inhabitants	State capitals ordered by percentage of the population residing in households with more than 2 people per bedroom.
Belém-PA	Macapá-AP
Manus-AM	Manaus-AM
Fortaleza-CE	Boa Vista-RR
Boa Vista-RR	São Luís-MA
São Lúis-MA	Reio Branco-AC
Macapá-AP	Belém-PA
Recife-PE	Porto Velho-RO
Rio Branco-AC	São Paulo-SP
Porto Velho-RO	Recife-PE
Vitória-ES	Cuibá-MT
Rio de Janeiro-RJ	Palmas-TO
Maceió-AL	Terezina-PI
São Paulo-SP	Rio de Janeiro-RJ
João Pessoa-PB	Fortaleza-CE
Salvador-BA	Natal-RN
Terezina-PI	Porto Alegre-RS
Aracaju-SE	Maceió-AL
Natal-RN	Brasília-DF
Palmas-TO	João Pessoa-PB
Goiânia-GO	Salvador-BA
Brasília-DF	Aracaju-SE
Cuiabá-MT	Curitiba-PR
Curitiba-PR	Campo Grande-MS
Belo Horizonte-MG	Belo Horizonte-MG
Porto Alegre-RS	Goiânia-GO
Florianópolis-SC	Florianópolis-SC
Campo Grande-MS	Vitória-ES

We assessed when the same capital is close in both columns and using the difference in positions that the same city occupies in the two columns as a metric. By also considering that the smaller this difference, the greater the adherence of the socioeconomic factor selected to classify and account for the population in the group that is unable to effectively reduce its rate of contagion, it is observed that the capital cities Fortaleza-CE, Vitória-ES, Palmas-TO, Cuiaba-MT and Porto Alegre-RS have a higher gap, pointing out that for these municipalities one should carefully assess the relationship between the selected socioeconomic factor and the spread of the disease in these municipalities. While for the other capital cities (more than 80% of the capital cities studied) the selected factor is presented as a relevant indicator for the disease to spread more quickly among the population.

### B. The Parameter Estimation

The estimation of the population percentage parameter, that does not effectively reduce its contamination rate, is carried out based on the data analysis previously described. The estimation of the parameters related to the evolution of the disease in the infected, on the other hand, can be initiated from the current literature on the new coronavirus, since it presents several studies related to the parameters necessary for the model. However, since some parameters may vary from study to study and this is probably due to characteristics of the population’s behavior. For example, as in some countries the number of daily contacts with other people is much lower than in other countries, it is necessary to adjust the model parameters to the reality of the scenario under analysis.

The estimation of these parameters can consider the numbers of confirmed cases and deaths observed over time, the estimation of underreporting, the prediction of the lethality rate according to the population’s age pyramid and the control measures adopted by the governments like closing non-essential activities and lockdown.

For the specific case studied in this article, values taken from the literature for parameters $\alpha $, $\gamma $ and $\omega $ were used, as shown in Table 3. We opted to use values from the literature because they have little variation. Next, it was necessary to estimate the parameters of contamination rates ($\beta _{a}$ and $\beta _{b}$) and to reduce this rate over time, which is the effect of control measures, for example, wearing a mask, hand hygiene and the products brought street, quarantine and social isolation.

**TABLE 3:** Simulation Parameters

Parameter	Value
$N$	2,292,758
$\delta $	0.8546
$\alpha $	1/2 day$^{-1}$
$\theta $	0.05
$\eta $	6.5
$\beta $	0.325
$\beta _{a}$	$\beta $. (1 - reduction rate)
$\beta _{b}$	$\beta $
$\gamma $	1/10 day${^{-}1}$
$\omega $	0.05

In the case study, to estimate these parameters, the data released by the Pará State Department of Public Health [46] for the period of the first 69 days after the first confirmed case was used. Thus, a brute force heuristic was used which, within a range of viable values according to the current literature and considering two decimal places for the aforementioned parameters, to minimize the error between the estimated values and the real values.

We evaluate the performance of the parameters estimation in terms of Mean Absolute Error (MAE) and Mean Root Square Error (RMSE) [47]. Mean Absolute Error and Mean Root Square Error are two of the most common metrics used to measure accuracy between the model predictions and actual data. In MAE all individual differences have equal weight, as expressed in (11). The value range varies from 0 to infinity and fewer score values show the goodness of learning models.

\begin{equation*} {\text {MAE}} = \frac {1}{n} \sum _{j=1}^{n}|y_{i} - \hat {y}_{i}|\tag{11}\end{equation*}

Here, $n$ is the sample size. Prediction errors can be understood as the distance from the best fit line and actual data points. In this sense, RMSE is a measure of how concentrated the actual data points are around the best fit line and can be calculated as:

\begin{equation*} {\text {RMSE}} = \sqrt {\frac {1}{n} \sum _{j=1}^{n}(y_{i} - \hat {y}_{i})^{2}}\tag{12}\end{equation*}

Finally, after parametrization, it is possible to perform a forecast for the epidemic of COVID-19 considering various scenarios. In this study, we consider the impact of different levels of non-pharmaceutical interventions implemented (including social distancing, quarantine of confirmed or suspected cases, hand hygiene). We represent the intervention within the simulation making assumptions about the impact of these interventions reducing the contamination rate.

### C. The Forecast for the Epidemic of COVID-19

In the last stage, the parametrized model is used to estimate the SARS-COV-2 spread in the time, by solving the system of ordinary differential equations. For this purpose, the LSODA algorithm (a variant of Livermore Solver for Ordinary Differential Equations) [48] was used. The algorithm was originally developed for FORTRAN and recently re-implemented in Python, in the SciPy library (library for scientific computing). During this stage, different values for reducing the contamination rate should be considered in view of the control measures adopted. In addition, the difference in results should be evaluated between the situation in which people in group $b$, who are unable to effectively reduce their contamination rate due to the consequences of social inequality, keep their contamination rate ($\beta _{b}$) constant throughout the period time and the situation where this rate similarly reduces the contamination rate of people in group $a$ ($\beta _{a}$). This assessment is essential in order to measure how much social inequality tends to aggravate the spread of the disease and that, thus, the necessary efforts can be devoted to mitigate this effect.

### D. A Case Study on the Deep Brazil

We developed a case study applying our model in a metropolitan region of Brazil located in the Amazon. Brazil is a continental country. Its territory is divided into 5 regions (North, Northeast, Midwest, Southeast and South), based on criteria, such as natural, social, cultural and economic aspects, by Brazilian Institute of Geography and Statistics. Inequalities between regions are enormous. The regions most affected by social problems are the North and Northeast, which have the worst Human Development Indexes in Brazil. Until June 17 2020, these regions had concentrated 55% of the cases of COVID-19 registered in the country according to the Ministry of Health [49]. The northern region is the largest in territorial extension and where most of the Brazilian Amazon is located. The proposed model was applied to an important part of the Amazon, the Metropolitan Region of Belém (MRB), in the State of Pará, consisting of 7 cities (Ananindeua, Belém, Benevides, Castanhal, Marituba, Santa Bárbara do Pará and Santa Isabel do Pará).

### E. Data

For Brazilian cities, the data are extracted from databases maintained by federal, state and municipal governments, such as National Household Sample Survey (PNAD), Brazilian Institute of Geography and Statistics (IBGE), Health Informatics Department of the Brazilian Ministry of Health (DATASUS) and COVID-19 cases reported by State Secretaries of Health.

For the case study, we extracted, from the most recent PNAD (2018), data on education, access to information and communication technology, general characteristics of domiciles and residents, professional information, average monthly income, etc., [12]. Residents data include gender, age group, color and race. Domicile data include type and condition of domiciles, material used on the walls, flooring, roof, basic essential sanitation, water supply, bathroom availability and sewage, garbage disposal, availability of electricity, furniture, stove, refrigerator, car, motor bike, etc.

From Ministry of Health [49] and the State Departments of Public Health, we obtained daily data on the number of confirmed cases, recovered cases and deaths. We assume that those sharing a single room with more than 3 people are not able to effectively isolate themselves and their probability of contact is indicated by parameter $\beta _{b}$ (infection rate of non-isolated infectious individuals). To estimate demand for health service, we used hospitalization rates (admittance and critical care) from previous analysis of COVID-19 in other countries, such as [1], [25], [38], [50] and [40]. Hospital and ICU beds were taken from National Records of Health Establishments [51] and DATASUS. From this data, we used the proposed model to estimate the number of infections and deaths, besides the demand for medical assistance in RMB towns and the impact of NPIs in mitigating effects of COVID-19 outbreak spread.

### F. Scenario

The Metropolitan Region of Belém (MRB) has a population (N) of 2,459,321 people according to [21]. The Metropolitan Region of Belém has 4,383 hospital beds, considering clinical, surgery and complementary (including ICUs) beds, an average of 1.78 beds per 1000 inhabitants, a figure below the national average of 1.95 according to Brazilian Federation of Hospitals (FBH) and National Confederation of Health (CNS) [52]. The world average is 3.2 [53]. The National Household Sample Survey (2018) was used for other demographic information. The number of confirmed, recovered and death cases were obtained from the Ministry of Health [49] and from the Pará State Department of Public Health (SESPA) [46].

Our studies on National Household Sample Survey (2018) showed that 14.54% of the population of MRB is not able to effectively reduce the contamination rate due to poor housing conditions, they do not have access to basic sanitation or live in homes with 3 or more people in the the same bedroom. Therefore, we consider $\delta $ of 85.46% (the percentage of the population can reduce their contamination rate). Other parameters used in our simulations were found in the recent literature according to clinical characteristics of 2019 novel coronavirus infection in China. The parameters were estimated using empirical assumptions, as cited before. Furthermore, we calibrated the parameters of the model to fit the reported data.

In the MRB, the first case was reported on March 18th, 2020 and until May 26th, 2020, 13,697 cases were confirmed. We used this series, with 69 days, to calibrate the model. On March 18th, activities were suspended by the local authorities in order to reduce the contamination rate. According to the Pará State Department of Public Health [46], from 10th day to 30th day, a reduction of 20% in contamination rate was observed. From 31st until the 69th day, the reduction rate was increased to around 50%. Therefore, after multiple fitting with data from [46], we determined: the incubation period was 2 days (range of 2 to 7) [40]. The rate of cases requiring ICU beds was 5% [40]. We consider $\gamma $ of 0.1 (range of 0.0721 to 0.238) and disease transmission probability ($\theta $) of 0.05 (range of 0.01 to 0.05) [54], [55]. We assumed a daily contact rate ($\eta $) of 6.5. We obtained a MAE of 182 and a RMSE of 295. Table 3 summarizes parameters used in the simulation.

## V. Results

**FIGURE 3.**
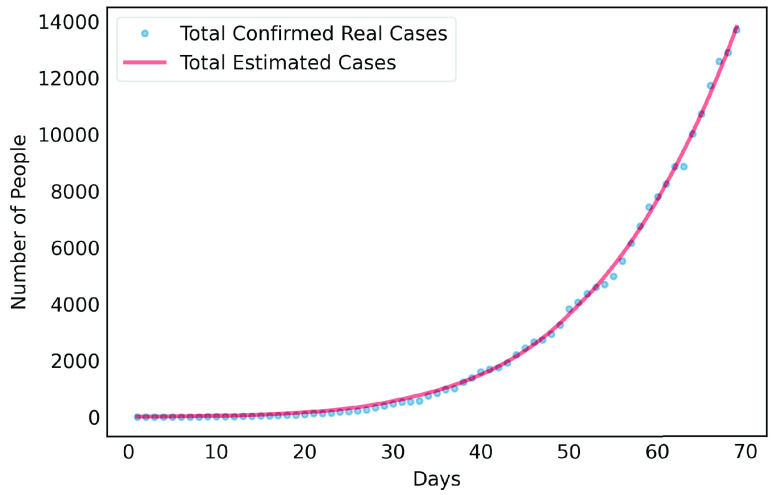
Predictions of the new SEIR model on the cumulative confirmed cases. Comparing real case vs. estimated.

Equations (6) to (10) denote in time t the respective number of susceptible, exposed, infectious and recovered cases, the latter summarizes all those that were removed because they became immune after recovery or who died due to the disease. Therefore, the number of infected, needing ICU beds and of deaths over time due to COVID-19 were estimated from our model. The input was based on analyses of scenarios including measures of NPI by the government, such as social distancing policies. In our model, the effect of these measures in the long term are represented as a coefficient (reduction rate) that adjusts the contamination rate $\beta $. The stricter the NPI measures, the greater the reduction rate, thus reducing the contamination rate $\beta _{a}$. Figure 3 presents the real versus estimated cases until May 26th based on the parameters of the model.

**FIGURE 4.**
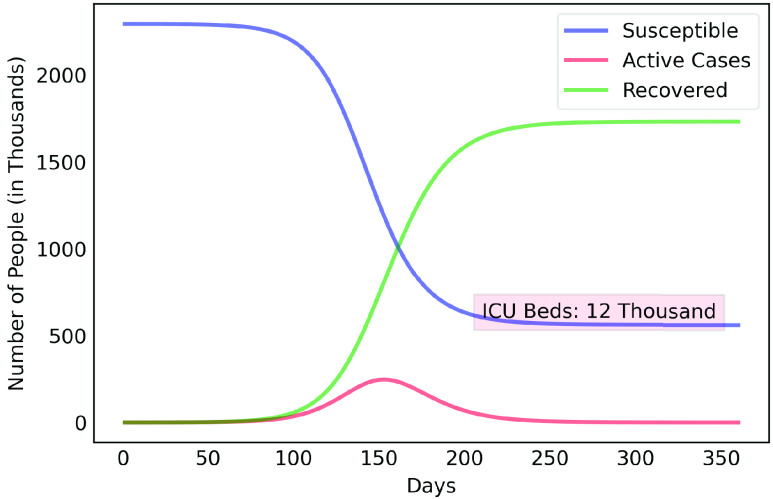
COVID-19 estimates for the Metropolitan Region of Belém considering 50% reduction rate of contamination.

After estimating the parameters to obtain results of Figure 3, we considered scenarios where contamination rate reduction, from the 70th day, was 20% to 85% for those infectious individuals in group $a$. This was done in order to compare the effect of different levels of reduction in contamination rate. For those people in group $b$, contamination rate was kept to the initial value. Figure 4 shows the results for the 50% reduction. Note that if the reduction in the contamination rate is maintained at this level, the peak of the contamination will be reached around the 150th day (August 14th, 2020). Considering a rate of 5% of confirmed cases require ICU beds, this would generate the need for 12 thousand ICU beds to meet the demand, well above what is currently available in this metropolitan region.

**FIGURE 5.**
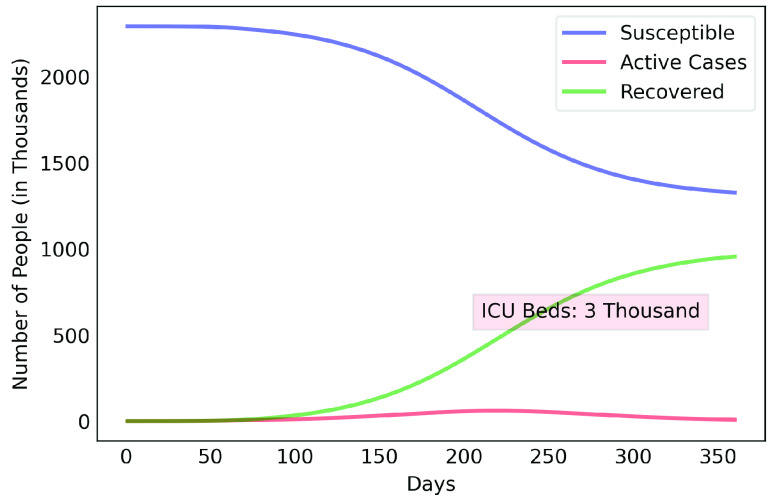
COVID-19 estimates for the Metropolitan Region of Belém considering 70% reduction rate of contamination.

Figure 5 shows that if the rate of contamination reduction rises to 70% and is maintained at that level, the contagion curve is flattened, shifting the peak to around the 225th day (October 28th, 2020), generating a demand for 3 thousand ICU beds, four times minus the expected demand for a 50% reduction in the contamination rate.

**FIGURE 6.**
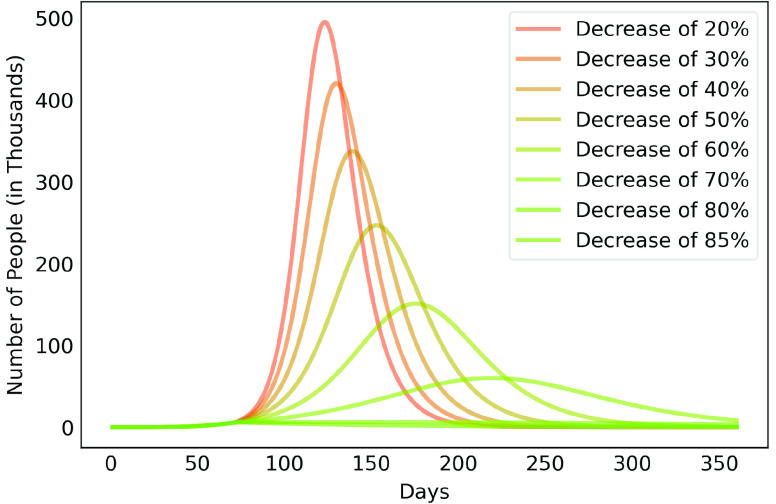
Active infected cases over time by reducing different contamination rates applied from the 70th day. Scenario with social inequality. 14.54% of the population cannot reduce their contamination rate due to issues such as overpopulated housing, basic sanitation problems, low income.

**FIGURE 7.**
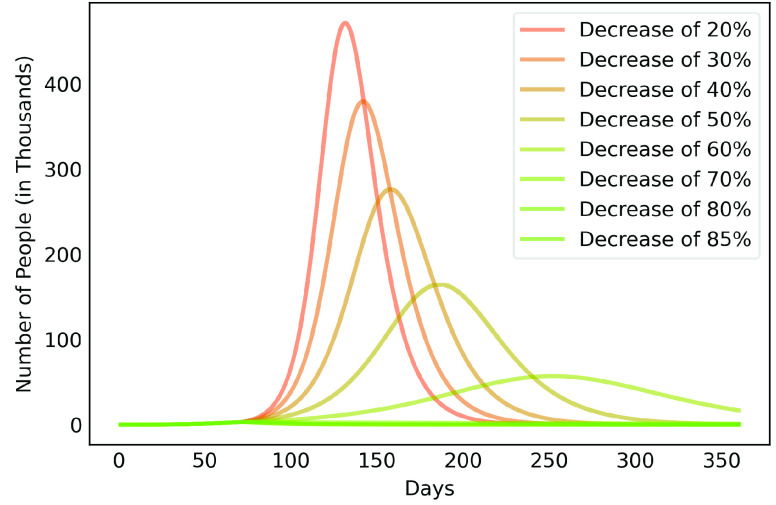
Active infected cases over time by reducing different contamination rates applied from the 70th day. Scenario with social equality, it considers that all the population is able to reduce their contamination rate.

**FIGURE 8.**
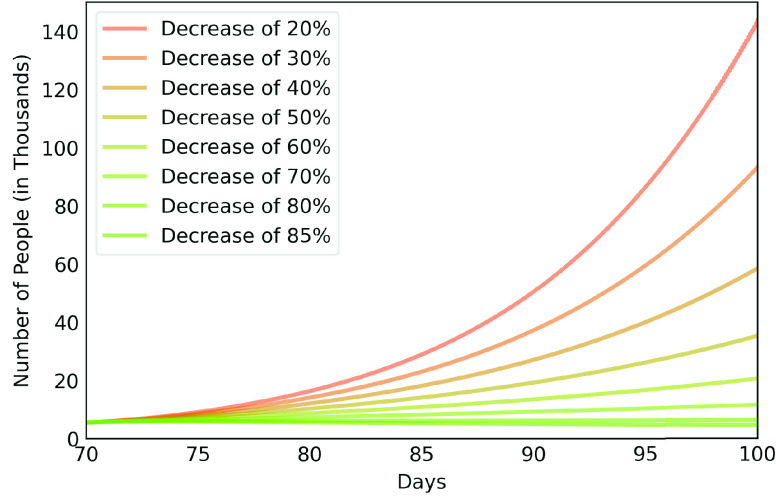
Active infected cases over time by reducing different contamination rates focusing on the next 30 days starting on the 70th day.

Figures 6, 7 and 8 show active infected cases over time by reducing contamination rate applied from the 70th day. In the model, the active cases are all individuals in compartment $I_{a}$ and $I_{b}$. For comparison purposes, the effect of being unable for everyone to reduce their contamination rate, Figure 6 considers that 14.54% of the population cannot reduce their contamination rate as they live in overpopulated households and with or without clean water. Thus, in this case, we consider $\delta $ as 85.46%. Figure 7 considers a scenario without social inequality in which all the population is able to reduce their contamination rate. Therefore, the curves below 50% of the reduction rate refer to relaxing the present NPI measures.

Observe that social inequality has a strong effect in the results. While reducing contamination rate by 70% in a real scenario considering social inequality (Fig. 6), there is still a peak of more than 55,000 active infected around the 225th day. In case without social inequality (usually considered in available studies), the same reduction rate reduces significantly the curve to numbers below 10,000 active infected throughout the estimated period. Fig. 8 highlights Fig. 6 focusing on the next 30 days starting on the 70th day. It shows that to keep the number of COVID-19 cases and the demand for beds at a manageable level, it is necessary to increase the reduction in the contamination rate to at least 60%. These results also demonstrate that premature relaxation of measure may lead to new outbreaks and as devastating as the first.

**FIGURE 9.**
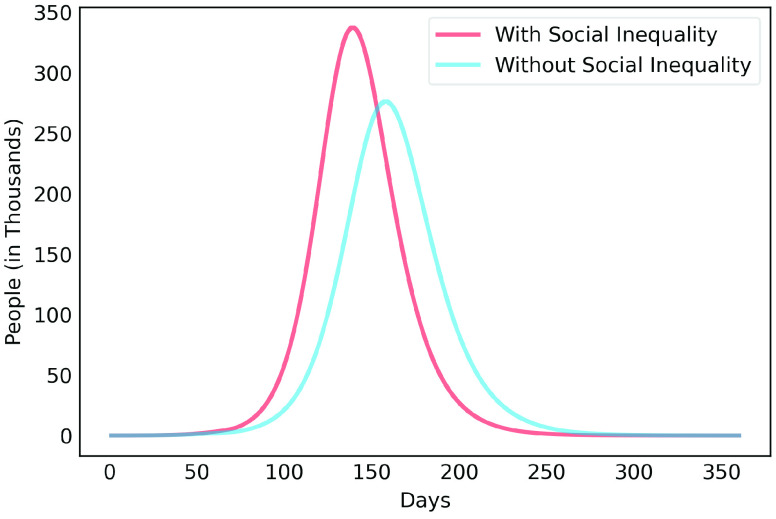
Active infected cases seen in a 1-year period for Metropolitan Region of Belém. Scenario with a 40% reduction in the contamination rate.

**FIGURE 10.**
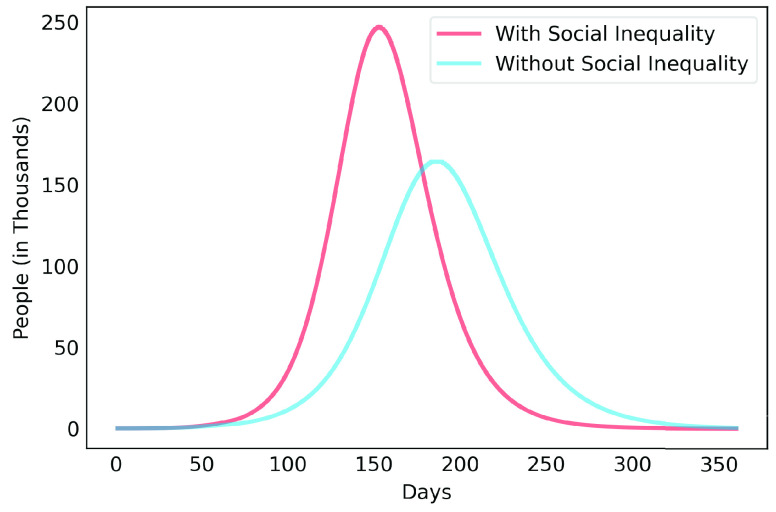
Active infected cases seen in a 1-year period for Metropolitan Region of Belém. Scenario with a 50% reduction in the contamination rate.

**FIGURE 11.**
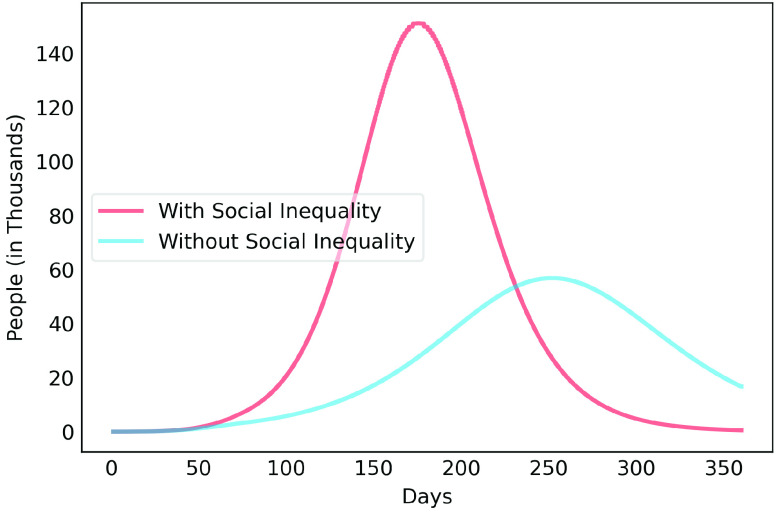
Active infected cases seen in a 1-year period for Metropolitan Region of Belém. Scenario with a 60% reduction in the contamination rate.

**FIGURE 12.**
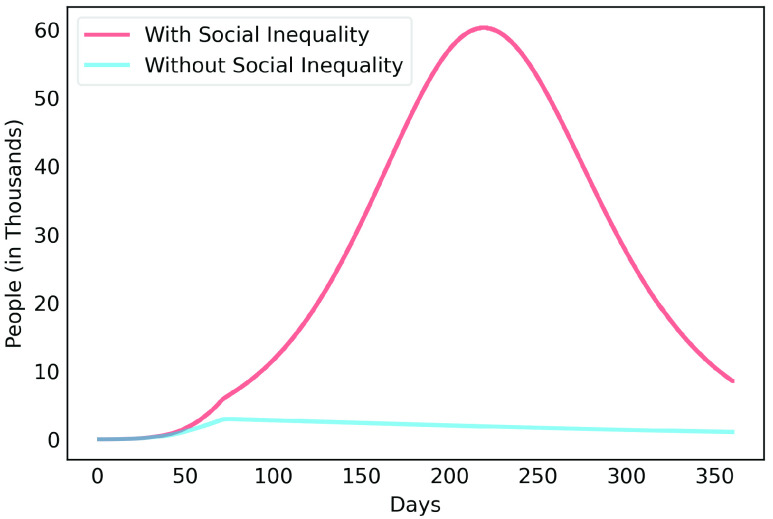
Active infected cases seen in a 1-year period for Metropolitan Region of Belém. Scenario with a 70% reduction in the contamination rate.

**FIGURE 13.**
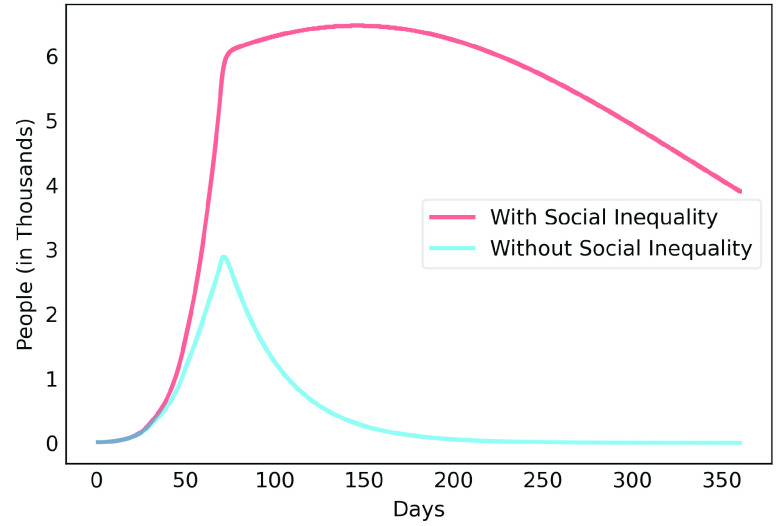
Active infected cases seen in a 1-year period for Metropolitan Region of Belém. Scenario with an 80% reduction in the contamination rate.

To highlight the difference, Figures 9, 10, 11, 12 and 13 show the impact of social inequality in the spread of COVID-19, in terms of the number of active infected cases considering different levels of non-pharmaceutical interventions, summarized in this study as a reduction in the contamination rate.

These results illustrate the disparity between the scenarios not only in the peak value of active infected, but also in terms of the day in which the curve achieves its maximum. Note that even considering strict interventions that lead to high rates of contagion reduction, social inequality has a strong effect on the propagation of the disease. For example, considering the case of a 70% reduction in the contamination rate, Metropolitan Region of Belém would have already experienced its peak of contagion if there were no social inequality. On the contrary, the escalation of the disease continues to rise, showing a peak 12 times greater than the previous case. Considering the 80% reduction, the more realistic case will have a peak twice as high as the scenario without social inequality.

It is worth mentioning that the differences in number of cases, the peak value and the day when the peak occurs may mislead the public administrators to make wrong decisions on which ones and with which intensity NPIs should be adopted. This shows the importance of making better estimates to the reality of each metropolitan region.

Our results show the impact of the social inequality on COVID-19 propagation considering the disparity of living conditions across the MRB population. The results presented are consistent with the concern of WHO in terms of low-capacity and humanitarian settings faced by some countries. For example, the document [56] draws attention to people living in collective sites are vulnerable to COVID-19 in part because of the health risks associated with movement or displacement, overcrowding, poor nutritional and health status among affected populations. The presented data for Brazil show that the simplest preventive measures against the new coronavirus, such as hand hygiene and social isolation, meet strong barriers in the Brazilian peripheries. Issues related to occupation, income, housing conditions, sanitation, access to water and public transport make up the worrying scenario that can become worse during the COVID-19 pandemic. These different aspects of socioeconomic status affect the capability of isolation/quarantine of populations in social vulnerability that depend on daily work for subsistence.

Regarding the pandemic in Brazil, another important issue to be discussed is underreporting, where the great majority of cases may not be diagnosticated and consequently unreported. The study [57] showed that pre-symptomatic transmission contributed to 48% and 62% of transmissions in Singapore and China (Tianjin data), respectively. Thus, there is an increasing risk that the actual number of infected people will be much higher than those notified by the control agencies. In Brazil, underreporting is mainly due to the low number of tests performed per million inhabitants and the delay in obtaining the results. According to the Worldometers Website [2], which compiles global data from COVID-19, Brazil has performed 1,709,468 tests to date (June 17th 2020), resulting in a number of 8,045 tests per million inhabitants, well below countries like Spain, Portugal and the United States, with 103,232, 98,705 and 78,832 tests per million inhabitants respectively.

In a pioneer study, called EPICOVID19-BR, leaded by the Federal University of Pelotas (UFPel) [58], the researchers interviewed and tested (for COVID-19) a significant sample of the population across more than 100 cities in different regions of Brazil (the most affected in the country). The objective was to estimate the number of contaminated cases for each city evaluated.

The first stage considered 133 cities from all the Brazilian states and took place between 14th and 21st May. Belém, the main city of MRB with 1,492,745 inhabitants, was included in the study and presented a percentage of 15.1% of people infected, which corresponds to 225,404 people, more than 20 times the number of officially confirmed cases to that date. A second phase of the research was carried out between June 4th and 7th. According to the researchers, the percentage of contaminated people in the city of Belém increased to 16.9% of the population, which corresponds to 252,273 people.

**FIGURE 14.**
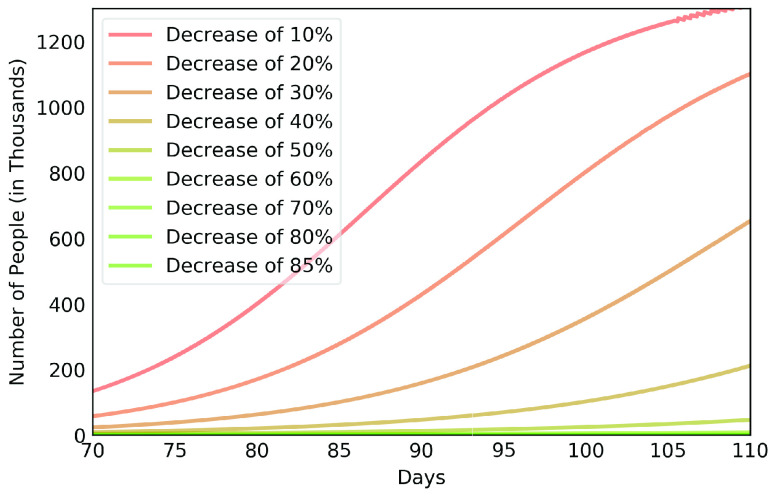
Total infected cases by reducing different contamination rate focusing on the next 40 days starting on the 70th day.

From the results of EPICOVID19-BR and in order to verify the real rate of contamination reduction experienced by Belém city, we made estimates of the total number of infected people over time considering the same initial contamination rate of MRB up to the 10th day of the series considered and different levels of contamination rate reduction. Figure 14 shows these results.

Observe that the first phase of the research occurred between days 78 and 85, while the second phase occurred between days 96 and 99. Therefore, Fig. 14 reveals that in the first phase the rate of contamination reduction was around 20% and increased to around 30% in the second phase of the research, below the 50% rate estimated by the authorities.

The COVID-19 lethality rate is affected by diverse situations, varying right from the age pyramid, access to healthcare units and percentage of people with serious diseases that might increase COVID-19 symptoms, etc. Table 4 shows the number of estimated deaths in a 1-year period for different levels of reduction on contamination rate and the Impact of different lethality rates. The World lethality rate (5.36%) was estimated according to Johns Hopkins Coronavirus resource center data for Global confirmed and deaths until March 03 2020 [59]. The lethality rate for Brazil (4.86%) was obtained from the Ministry of Health [49]. Because China is an important reference, we also compared it with the lethality rate obtained in the study [60], 1.38%. We also compared the total number of deaths using estimates from studies that considered underreporting in Brazil. Reference [66] estimates under-reporting and lethality rates for each state in Brazil according to age. In the study, the lethality rate for the State of Pará is estimated at 0.68%. Finally, we used the result of the study on underreporting conducted by UFPEL [58] and the number of deaths officially reported by the Pará State Department of Public Health [46] to estimate the most realistic lethality rate for the city of Belém, which is responsible for more than 50% of cases in the MRB. All the values in Table 4 are calculated considering the social inequality scenario.

**TABLE 4:** Impact of Different Lethality Rates on thE Total Number of Deaths Seen in a 1-Year Period

Reduction in contamination rate (%)	Total number of infected people (thousand)	World (5.36%) (thousand)	Brazil (4.86%) (thousand)	Lethality rates China (1.38%) (thoused)	State of Pará (0.68%) (thoused)	Belém City (0.7%) (thoused)
20	2100	112.56	102.06	28.98	13.23	14.7
30	2020	108.272	98.172	27.876	12.726	14.14
40	1910	102.376	92.826	26.358	12.033	13.37
50	1730	92.728	84.078	23.874	10.899	12.11
60	1450	77.72	70.47	20.01	9.135	10.15
70	956	51.24	46.46	13.19	6.02	6.69
80	174	9.32	8.45	2.40	1.09	1.21
85	63.7	3.41	3.09	0.87	0.40	0.44

As shown in Table 4, in a more pessimistic case (the world lethality rate), the worst scenario leads to 112.56 thousand deaths, when there is 20% reduction of contamination and drastically falls to 3.41 thousand reducing to 85%. Based on Brazilian Ministry of Health data (until March 18th 2020), MRB can reach 84.078 thousand deaths if the 50% reduction in the contamination rate is maintained for 1-year period. To date (June 18th 2020), the Pará State Department of Public Health [46] accounts for 2380 deaths in the MRB. By applying the same lethality rate of China to MRB, the results reach 13.19 thousand deaths if reduction in the contamination rate increases from 50 to 60% and is maintained at this level.

As already mentioned, the official numbers are under the effect of underreporting and, therefore, reported mortality rates may be higher than real rates. For this reason, we believe that the estimated lethality rates for the state of Pará and the city of Belém, considering the underreporting of cases, present numbers much closer to reality. From Table 4 we can infer that it is necessary to increase the rigor of the non-pharmaceutical intervention measures currently implemented in MRB in order to minimize the effects of COVID-19 on the number of deaths. Table 5 presents the impact of reduction of contamination rate in terms of demand for ICU beds seen in a 1-year period.

**TABLE 5:** Impact of Different Reduction Contamination Rate on the Demand for ICU Beds Seen in a 1-Year Period

Reduction in contamination rate (%)	Peak Number of Active Infected (thousand)	Peak ICU Beds (thousand)
20	495	24.8
30	420	21
40	337	16.9
50	246	12.3
60	151	7.6
70	60.26	3.0
80	6.47	0.32
85	5.89	0.29

Note that to keep the demand for ICU beds at a manageable level within the current reality of MRB’s health infrastructure, it is necessary to increase the reduction in the contamination rate to at least 70%. Anything below that already implies a collapse in the health system soon. It is worth mentioning that efforts to reduce the rate of contamination are made to flatten the disease evolution curve in order to allow health infrastructure to be able to meet the demand of patients and allow time to develop efficient treatment protocols and vaccines.

In addition, the efficiency of the measures is closely linked to adherence by the population and also to the living conditions in which this population is inserted. Therefore, governments must adopt special measures for those who live in precarious housing conditions and can allow these people to be more protected as well and to be able to protect their peers. The reduction in the rate of contamination is not only affected by social distancing or restricted mobility, but also by the tracking and control of suspected cases.

Finally, we should consider that the problem of underreporting is not unique to Brazil. Most surveillance and notification systems are affected by a degree of underestimation [61]. In general, this occurs because not all cases seek healthcare (a portion of those infected is asymptomatic or has mild symptoms of the disease) and the healthcare system can fail to adequately report symptomatic cases that have sought medical advice [61]. There are several methods to estimate the underreporting, but this analysis is not in the scope of this work.

We should note that our new SEIR modelling approach did not take into account some aspects, such as the age-specific contact rates, the effect of the age distribution over the population and the probability rate of becoming susceptible again, after having already recovered from the infection. Besides, the forecast model as it is currently implemented does not differentiate between notified and underreported cases, which does not prejudice the analysis of the results obtained, since the main objective of the article is to show the impact of social inequality on the spread of COVID-19 in the long term. This characteristic, however, can be included in future work by associating the model with other strategies, such as analytical models and based on artificial intelligence and models based on tests in a representative sample of the population, in order to provide estimates of the actual percentage of infected, allowing more accurate calculations of the lethality rate of the disease, which in an inverse analysis, can provide more accurate values of the total number of infected. Our model is fully flexible and suited to include, in future works, new analyzes in order to mitigate the current limitations and make it more robust.

## VI. Limitations of the Study

The model and the results described in this paper show how it is important to consider economic and social characteristics about the population under study when predicting the spread of infectious diseases like COVID-19. For countries with high social inequality, the predictions results can be underestimated since the precarious living conditions are an obstacle to enforce and implement NPIs.

For the Brazilian case study, the main characteristic considered in the model was the proportion of people living in houses with high demographic density per room and inadequate basic sanitation services. However, other characteristics can also influence in the spread of the disease, such as, number of informal works.

So, the limitations of this paper are related to study what other characteristics should be included for Brazil or for other countries. Also, a detailed study about how these characteristics influence in the results of government actions like lockdown should be conducted. The main difficulty to overcome these limitations is to obtain actual data.

## VII. Conclusion

In this study, a has been proposed for characterizing the impact of social inequality on COVID-19 propagation, especially noticed in developing countries.

Our analysis for Brazil suggests that housing conditions are a powerful determinant in the incidence of cases and deaths as a result of COVID-19 and therefore social conditions are of fundamental importance in predicting the spread of disease. Otherwise, this prediction can be underestimated, impacting the efficiency of the measures implemented.

Our approach was based on the living conditions of the population, considering that individuals who live in overpopulated houses and with difficulty in accessing basic sanitation and clean water are unable to carry out the recommendations for social distancing and hygiene even though they are in home quarantine and, therefore, fail to decrease their rate of contamination. This is the reality for a significant portion of the population in developing countries, such as Brazil and India.

Our model tries to minimize the deficiency of SEIR regarding this social inequality by combining this model with demographic data. The estimated impacts regarding the number of infected and fatalities are compared in the presence of non-pharmaceutical interventions, represented in the model as a coefficient that adjusts the contamination rate of the portion of the population that is able to carry out sanitary and social distancing recommendations.

A case study was performed for the Deep Brazil, specifically for the Metropolitan Region of Belém (MRB), State of Pará, located in the extreme north of Brazil. Our results suggest that social inequality has a strong impact on the number of cases of COVID-19, increasing its incidence in the population and changing the dates of outbreaks. In addition, the results illustrated how crucial are the NPIs measures to break the disease transmission chains, thus minimizing their effects in health infrastructure in terms of ICU beds.

Note that results presented in this study provide long-term estimates and show the impact of social inequality on the spread of COVID-19. Our focus is not to measure the effect of a specific measure in the short term, but to measure how much the scenario worsens considering social inequality. Therefore, this factor must be considered in the decision making by authorities to manage the public health crisis. To evaluate the effect of specific measures in the short term, it is necessary to associate the model with other strategies, such as modifying the model parameters in specific periods of time in order to simulate social distance policies (for example, closing schools, remote working, lockdown).

It is noteworthy that, although this study addresses the situation of developing countries that have a high degree of inequality, this study can also be applied to developed countries that have a high rate of social inequality, such as the United States. For countries or locations with a low rate of social inequality, the present study tends to present the same results as traditional models.

Finally, this study shows that tools, such as the model proposed here, play a major role to scientifically understand the impacts of the COVID-19 in order to properly make necessary decisions for containment of its impacts. In our evaluation, we have not considered costs of the mitigation measures, but we understand that they will definitely and strongly impact the economies of all the countries that were hit by the disease, in particular, those with low and medium income. These will not only suffer from the health crisis, but also have to face a social and economic crisis during the next few years.
